# Comparison of ARIMA and LSTM for prediction of hemorrhagic fever at different time scales in China

**DOI:** 10.1371/journal.pone.0262009

**Published:** 2022-01-14

**Authors:** Rui Zhang, Hejia Song, Qiulan Chen, Yu Wang, Songwang Wang, Yonghong Li

**Affiliations:** 1 Chinese Center for Disease Control and Prevention, Beijing, China; 2 National Institute of Environmental Health, Chinese Center for Disease Control and Prevention, Beijing, China; Instituto Nacional de Astrofisica Optica y Electronica, MEXICO

## Abstract

**Objectives:**

This study intends to build and compare two kinds of forecasting models at different time scales for hemorrhagic fever incidence in China.

**Methods:**

Autoregressive Integrated Moving Average (ARIMA) and Long Short-Term Memory Neural Network (LSTM) were adopted to fit monthly, weekly and daily incidence of hemorrhagic fever in China from 2013 to 2018. The two models, combined and uncombined with rolling forecasts, were used to predict the incidence in 2019 to examine their stability and applicability.

**Results:**

ARIMA (2, 1, 1) (0, 1, 1)_12_, ARIMA (1, 1, 3) (1, 1, 1)_52_ and ARIMA (5, 0, 1) were selected as the best fitting ARIMA model for monthly, weekly and daily incidence series, respectively. The LSTM model with 64 neurons and Stochastic Gradient Descent (SGDM) for monthly incidence, 8 neurons and Adaptive Moment Estimation (Adam) for weekly incidence, and 64 neurons and Root Mean Square Prop (RMSprop) for daily incidence were selected as the best fitting LSTM models. The values of root mean square error (RMSE), mean absolute error (MAE) and mean absolute percentage error (MAPE) of the models combined with rolling forecasts in 2019 were lower than those of the direct forecasting models for both ARIMA and LSTM. It was shown from the forecasting performance in 2019 that ARIMA was better than LSTM for monthly and weekly forecasting while the LSTM was better than ARIMA for daily forecasting in rolling forecasting models.

**Conclusions:**

Both ARIMA and LSTM could be used to build a prediction model for the incidence of hemorrhagic fever. Different models might be more suitable for the incidence prediction at different time scales. The findings can provide a good reference for future selection of prediction models and establishments of early warning systems for hemorrhagic fever.

## Introduction

Hemorrhagic fever, a zoonotic viral infection caused by Hemorrhagic fever viruses is still a serious threat to humans. The hemorrhagic fever viruses have great potential risk because of extreme pathogenicity and potential for transmission by fine particle aerosol [1]. Humans become infected through the bites of ticks, by contacting with a patient with hemorrhagic fever and by contacting with blood or tissues from viremic livestock [2]. The disease has developed into a serious public health concern. The incidence of hemorrhagic fever ranks in the top ninth among the category A, B and C infectious diseases in China. The reported incidence rate of hemorrhagic fever was 8.2 cases per million in China in 2017 [3].

In recent years, many studies have developed prediction models of the incidence of hemorrhagic fever. These relevant researches mainly used particular popular methods, such as Autoregressive Integrated Moving Average (ARIMA) [4], logistical regression model [5], Seasonal Autoregressive Fractionally Integrated Moving Average (SARFIMA) [6], and Seasonal Difference Space-Time Autoregressive Integrated Moving Average (SD-STARIMA) [7]. With the rapid development of neural network, many algorithms have been used for prediction analysis [8–10]. However, there are few studies on predicting the incidence of hemorrhagic fever based on neural network. Therefore, whether the neural network model is better than the traditional models in predicting the incidence of hemorrhagic fever is still unknown.

For diseases that occur in cyclic or repeating patterns, time series models have been used to predict future outbreaks. ARIMA is a traditional time series model, which is one of the most popular methods used in infectious disease prediction, such as hemorrhagic fever [11], COVID-19 [12], brucellosis [13], hepatitis [14, 15], syphilis [16], influenza [10, 17], tuberculosis [18, 19], HIV [20], as well as blood glucose concentrations and hypoglycemia [21], hospital daily outpatient visits [22], and so on. Although ARIMA model has become a standard tool for time series, it has two disadvantages. Firstly, it assumes that the relationship between independent variables and dependent variables is linear. Secondly, ARIMA model assumes that the standard deviation of error with time is constant [10]. However, the relationship in the real world is more complex than the assumption in the model. Therefore, when the data structure is complex, the performance of ARIMA model is often poor. Long Short-Term Memory Neural Network (LSTM) model is a neural network that accounts for dependencies across observations in a time series. It is a novel recurrent network architecture in conjunction with an appropriate gradient-based learning algorithm. LSTM is designed to overcome these error back-flow problems. It can learn to bridge time intervals in excess of 1000 steps even in case of noisy, incompressible input sequences, without loss of short time lag capabilities [23]. LSTM model has been increasingly used in recent years to forecast in many fields such as traffic flow prediction [24], speech recognition [25] as well as disease prediction [20, 26, 27]. Both ARIMA and LSTM are suitable for time series prediction. However, most of the previously reported studies on diseases prediction using ARIMA and LSTM were based on monthly data [11, 14–16, 19–21]. Several studies were based on weekly data [17, 27] or daily data [10, 21, 22]. As far as we know, no studies have compared the prediction models based on data at different time scales.

In this study, we plan to adopt ARIMA and LSTM to build prediction models for the incidence of hemorrhagic fever in China based on the monthly, weekly and daily incidence data from January 2013 to December 2018 and then compare the forecasting performance using the data from January to December 2019. The model building and comparison intends to provide suggestions, act as a reference for those choosing the best prediction models in future studies, aid the development of early warning systems for hemorrhagic fever control and prevention.

## Materials and methods

### Hemorrhagic fever data

The daily national incidence data of hemorrhagic fever in China from January 2013 to December 2019 was applied from the official website of the Public Health Science Data Center. The total dataset used in this study consisted of 84 months, 365 weeks and 2,557 days.

### Algorithms

The ARIMA and LSTM models developed for forecasting the time series are combined with “Rolling Forecasting Origin”. The rolling forecasting origin focuses on a single forecast that the next data point to predict for each data set. This approach uses training sets, each one containing one more observation than the previous one and uses this to look ahead one step in time. In general, a rolling forecast uses the latest data to forecast the next time step. There are several variations of rolling forecast: One-step or multi-step without re-estimation and multi-step forecast with re-estimation. In this study, the variations of one-step forecast with re-estimation was combined with the two models. Data on hemorrhagic fever incidence cases from January 2013 to December 2018 were used to build ARIMA and LSTM models. The data from January to December in 2019 were used to evaluate the forecasting performance of these models.

### ARIMA model

ARIMA is a class of models that captures temporal structures in time series data with a linear regression based forecasting approach. Therefore, it is best for one-step out-of-sample forecasting, also known as a rolling forecasting. The model is re-fitted to build the best estimation model for each step. ARIMA has Autoregressive (AR) and Moving Average (MA) components and Seasonal Autoregressive Integrated Moving Average (SARIMA) also has a seasonal version of these in addition. The model is expressed as ARIMA (p, d, q) generally, p means the order of auto-regression, d means the degree of trend difference and q means the order of moving average [14, 22, 28].

The modeling process of ARIMA can be divided into three stages: time series stability, parameter estimation and model evaluation. In the first stage, the Augmented Dickey-Fuller (ADF) unit-root test is used to estimate whether the time series is stationary or not. Log transformation and differences are preferred ways to stabilize the time series [22]. Seasonal differences were adopted to stabilize the term trend and periodicity in this study. In the second stage, Autocorrelation function (ACF) graph and partial autocorrelation (PACF) graph are used to estimate parameters [19]. Automatic identification and artificial estimation were adopted in this study. “auto.arima()” command in R software was first adopted to automatically identify the model parameters. Then ACF, PACF and differences were employed to identify p, d, q and P, D, Q. In the third stage, Q-Q plots are used to tests whether the model’s residuals meet an independent normal distribution. All the models that passed the Box-Ljung test (show a white noise sequence) are compared using Akaike information criterion (AIC) so that the best model can be found [21], usually with the lowest AIC value. In this study, we used the incidence of hemorrhagic fever from January 2013 to December 2018 to build and test the ARIMA model.

### LSTM model

LSTM is a special type of Recurrent Neural Network (RNN) with the capability of remembering the values from earlier stages for the purpose of future use and it has proved useful for time series forecasting [20, 27].

LSTM Deep Learning algorithm, developed by Hochreiter and Schmidhuber (1997), allows the preservation of the weights that are forward and back propagated through layers. The network can continue to learn over many time steps by maintaining a more constant error. Thus, the network can be used to learn long-term dependencies. Adaptive Moment Estimation (Adam), Stochastic Gradient Descent (SGD) and Root Mean Square Prop (RMSProp) optimizers are excellent general-purpose optimizers that perform gradient descent via backpropagation through time [29].

LSTM networks try to combat the vanishing/exploding gradient problems by introducing gates and an explicitly defined memory cell. These are inspired mostly by circuitry, not by so much by biology. Each neuron contains one memory cell and three gates: input, output and forget [30]. The function of these gates are to safeguard the information by stopping or allowing the flow of it. The input gate determines how much of the information from the previous layer gets stored in the cell. The output layer takes the job on the other end and determines how much the next layer gets to know about the state of this cell. The forget gate is useful to forget some prior values, i.e., it controls the extent to which a value remains into the cells due to some future works.

The training of the LSTM model can be divided into the following three stages. In the first stage, because the LSTM models are sensitive to the scale of the input data, the data is rescaled and normalized to the range of 0 to 1. In the second stage, the time step is set to 7/30/60, which means using the data of the previous 7/30/60 months/weeks/days to predict the incidence of the next month/week/day. In the third stage, one hidden layer is set for the LSTM model with neurons options of 4/8/16/32/64/72/128/256 and the optimization functions of Adam/SGD/RMSProp. All these learning processes run in 200/250/500/1000 epochs. The initial learning rate was determined to be “0.005” and instructed the model to drop the learning rate every 125 epochs by multiplying by 0.2. Based on the above results, we choose the optimal model according to the minimum root mean square error (RMSE) of the validation set.

### Forecast accuracy access

Three indexes were employed in accessing model fitting and forecasting efficiency: RMSE, mean absolute error (MAE) and mean absolute percentage error (MAPE) [2, 14]. These three indexes are defined as:

$$RMSE=\sqrt{\frac{\sum_{i=1}^{n}{\left(X_{i}-{\hat{X}}_{i}\right)}^{2}}{n}}$$

$$MAE=\frac{\sum_{i=1}^{n}\left|X_{i}-{\hat{X}}_{i}\right|}{n}$$

$$MAPE=\frac{\sum_{i=1}^{n}\frac{\left|X_{i}-{\hat{X}}_{i}\right|}{X_{i}}\times 100}{n}$$

*X_i_* is the actual value, ${\hat{X}}_{i}$is the predicted value, *i* = 1…*n* and *n* is the number of observation.

### Data and analysis

Excel 2016 was used to build the database of monthly, weekly and daily incidence of hemorrhagic fever in China and R 3.6.2 software was adopted to develop the ARIMA model and LSTM model. For all statistical tests, statistical significance was a two-tailed *P*<0.05.

## Results

### Trends of hemorrhagic fever in China

**Fig 1:**
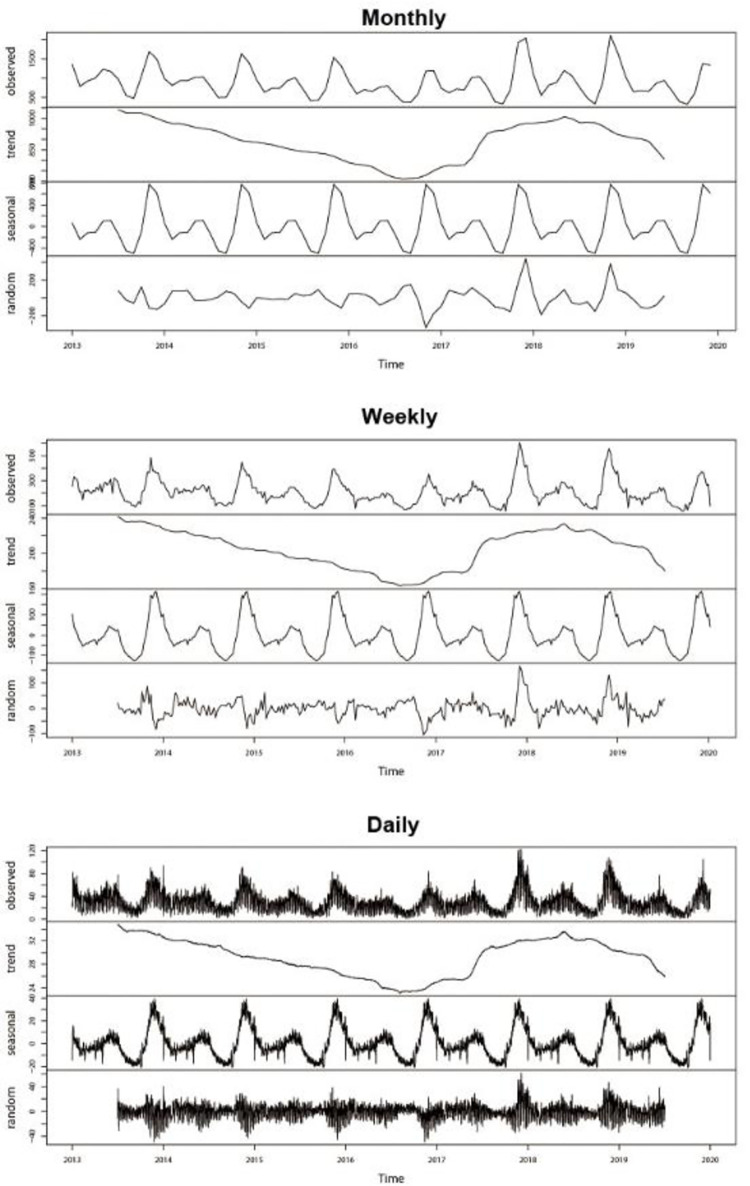
Decomposition of additive time series of the monthly, weekly and daily incidence of hemorrhagic fever in China from January 2013 to December 2019.

A total of 75,144 hemorrhagic fever cases were enrolled during 2013 to 2019. The average daily number of hemorrhagic fever was 29 cases with a minimum daily number of 1 case and the maximum daily number of 98 cases. Fig 1 shows the decomposition analysis for additive time series for the monthly, weekly and daily incidence cases of hemorrhagic fever in China from January 2013 to December 2019. These included the trend of the observed cases, the long-term and seasonal trends and the random variation. The long-term trend showed that the overall incidence of hemorrhagic fever in China presented a downward trend from 2013 to 2017, followed by a slowly rising trend in 2018 and then decreasing in 2019. The seasonal component showed strong seasonality. The random component shows the randomness of the data. These indicated that the dataset of the incidence of hemorrhagic fever during 2013 to 2019 in China is a typical time series.

### Fitting models with ARIMA

**Fig 2:**
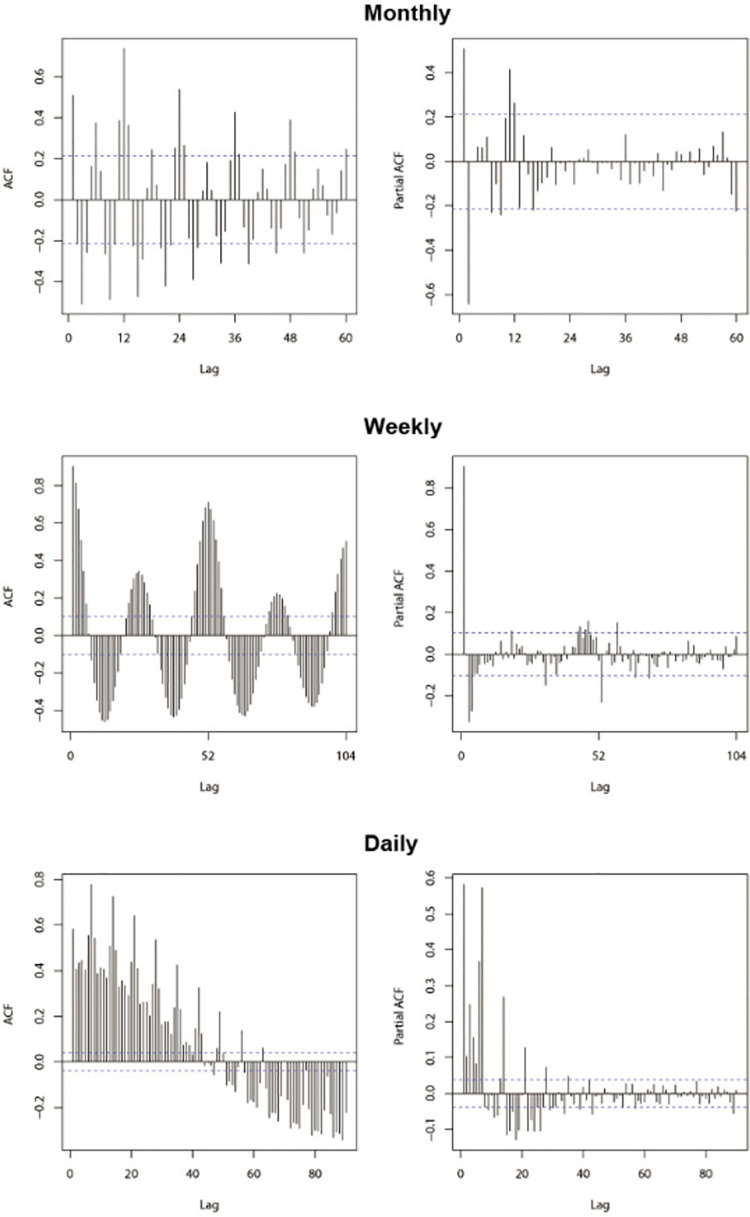
The ACF graph and PACF graph of monthly, weekly and daily hemorrhagic fever incidence series.

The incidence data of hemorrhagic fever in China from January 2013 to December 2018 was used as a training dataset to build prediction models. The first trend difference (d = 1) and seasonal difference (D = 1) were used to eliminate numerical instabilities in the monthly and weekly time series. The daily incidence data from 2013 to 2018 showed a basically stationary trend with time, thus d = 0. The result of ADF test after the difference showed statistical significance with *P* = 0.02 for monthly data and *P* = 0.01 for weekly data. Then the ACF graph and PACF graph (Fig 2) were done to help estimate the parameters of ARIMA model.

During 1 cycle, The ACF declined to 0 after lag 1 or lag 3 and the PACF was at lag 2, thus p = 1 or 3, q = 2. During 2 circles, the ACF declined at the end of the first circle (lag 12) but close to 0, thus Q = 1 or 2. The PACF was 0 at lag 12, thus P = 0 or 1 in the monthly incidence series. Under the same principle, parameters from the other models were estimated by the graphs of weekly and daily incidence series. In addition, another five models at each time scale were primarily selected for further model selection by running “auto.arima()” in R 3.6.2 software to recognize parameters automatically. The results of Box-Ljung test and AIC values of different models were shown in Table 1.

**Table 1:** Box-Ljung test and AIC for different models of monthly, weekly and daily time scales.

Model			Box-Ljung test		AIC
			χ^2^	*P*-value	
**Monthly**	**Identification**	ARIMA (3, 1, 2) (0, 1, 1)_12_	0.02	0.90	764.62
	**Auto.arima**	**ARIMA (2, 1, 1) (0, 1, 1)**_**12**_	0.33	0.56	**763.74**
		ARIMA (1, 1, 1) (0, 1, 1)_12_	0.90	0.34	772.67
		ARIMA (2, 1, 1) (1, 1, 1)_12_	0.27	0.60	768.62
		ARIMA (2, 1, 2) (1, 1, 2)_12_	0.42	0.51	764.51
		ARIMA (3, 1, 1) (0, 1, 1)_12_	0.14	0.70	765.22
**Weekly**	**Identification**	**ARIMA (1, 1, 3) (1, 1, 1)**_**52**_	0.02	0.88	**2584.64**
	**Auto.arima**	ARIMA (0, 1, 1) (1, 1, 1)_52_	0.01	0.95	2599.13
		ARIMA (2, 1, 2) (1, 1, 1)_52_	0.02	0.87	2586.75
		ARIMA (1, 1, 3) (1, 1, 0)_52_	0.02	0.89	2590.08
		ARIMA (1, 1, 3) (1, 1, 2)_52_	0.03	0.88	2586.82
		ARIMA (0, 1, 3) (1, 1, 1)_52_	0.31	0.57	2600.03
**Daily**	**Identification**	ARIMA (4, 0, 2)	0.03	0.85	20420.38
	**Auto.arima**	**ARIMA (5, 0, 1)**	0.11	0.73	**20379.97**
		ARIMA (5, 0, 2)	0.05	0.82	20421.46
		ARIMA (4, 0, 1)	0.53	0.46	20458.65
		ARIMA (5, 0, 0)	2.32	0.12	20826.53
		ARIMA (4, 0, 0)	0.39	0.53	20842.21

**Fig 3:**
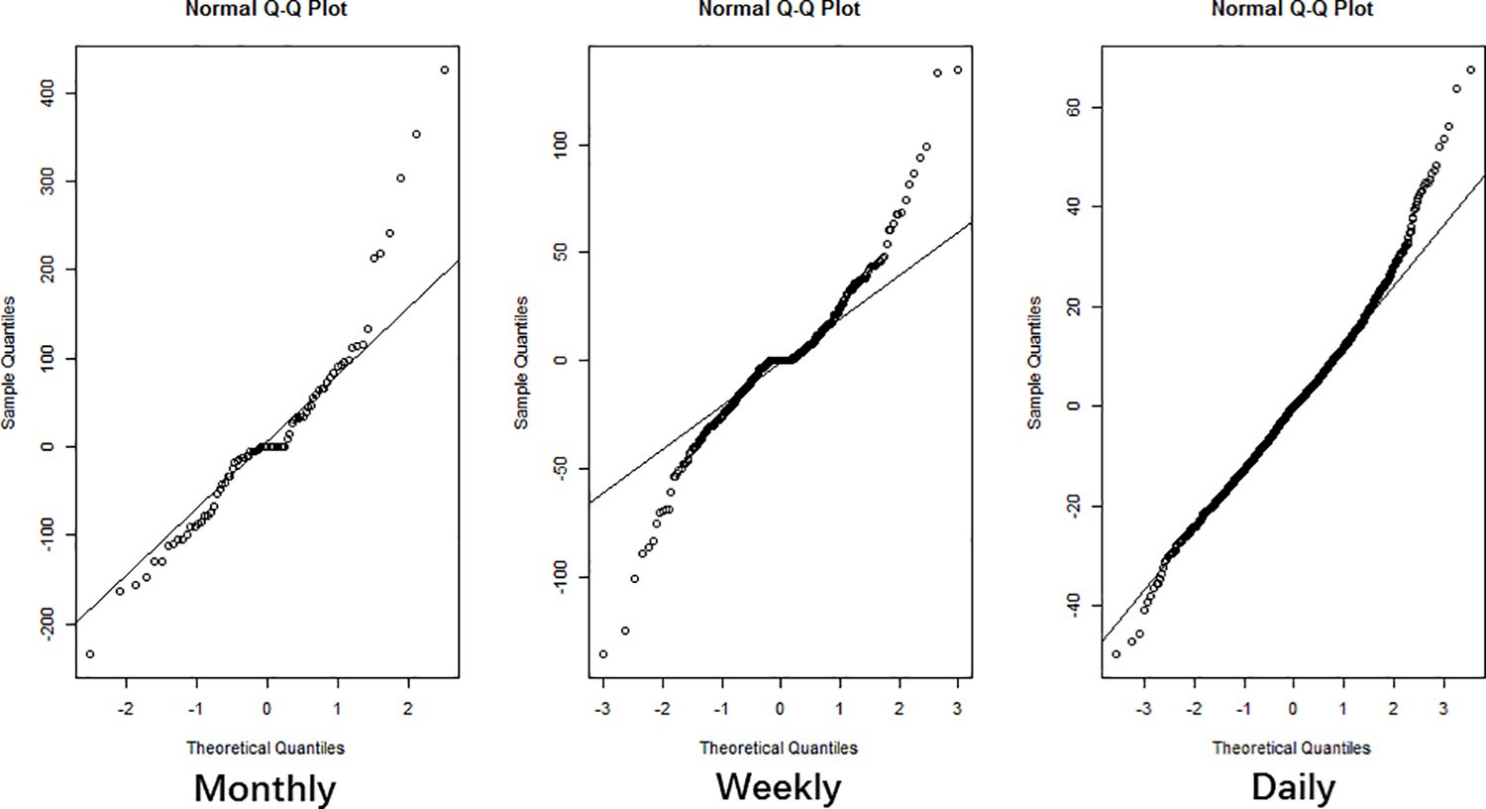
The normal Q-Q plot of monthly, weekly and daily residuals.

According to Table 1, all models meet the requirement of white noise of residuals time series (*P*>0.05) so the AIC values were compared. Automatically, the recognized model ARIMA (2, 1, 1) (0, 1, 1)_12_ had the lowest AIC and was selected as the best ARIMA model in the monthly incidence series. Model ARIMA (1, 1, 3) (1, 1, 1)_52_ for weekly incidence and ARIMA (5, 0, 1) for daily incidence were selected by the same reason. The Q-Q plots for all three selected fitting models showed that the residuals satisfied an independent normal distribution (Fig 3) which indicated that the fitting models were effective.

### Fitting models with LSTM

The results showed that the model with 64 neurons and SGDM for monthly incidence, 8 neurons and Adam for weekly incidence, and 64 neurons and RMSprop for daily incidence were the best fitting models which had the lowest RMSE in comparison with the models using other parameters.

### Model comparison

**Fig 4:**
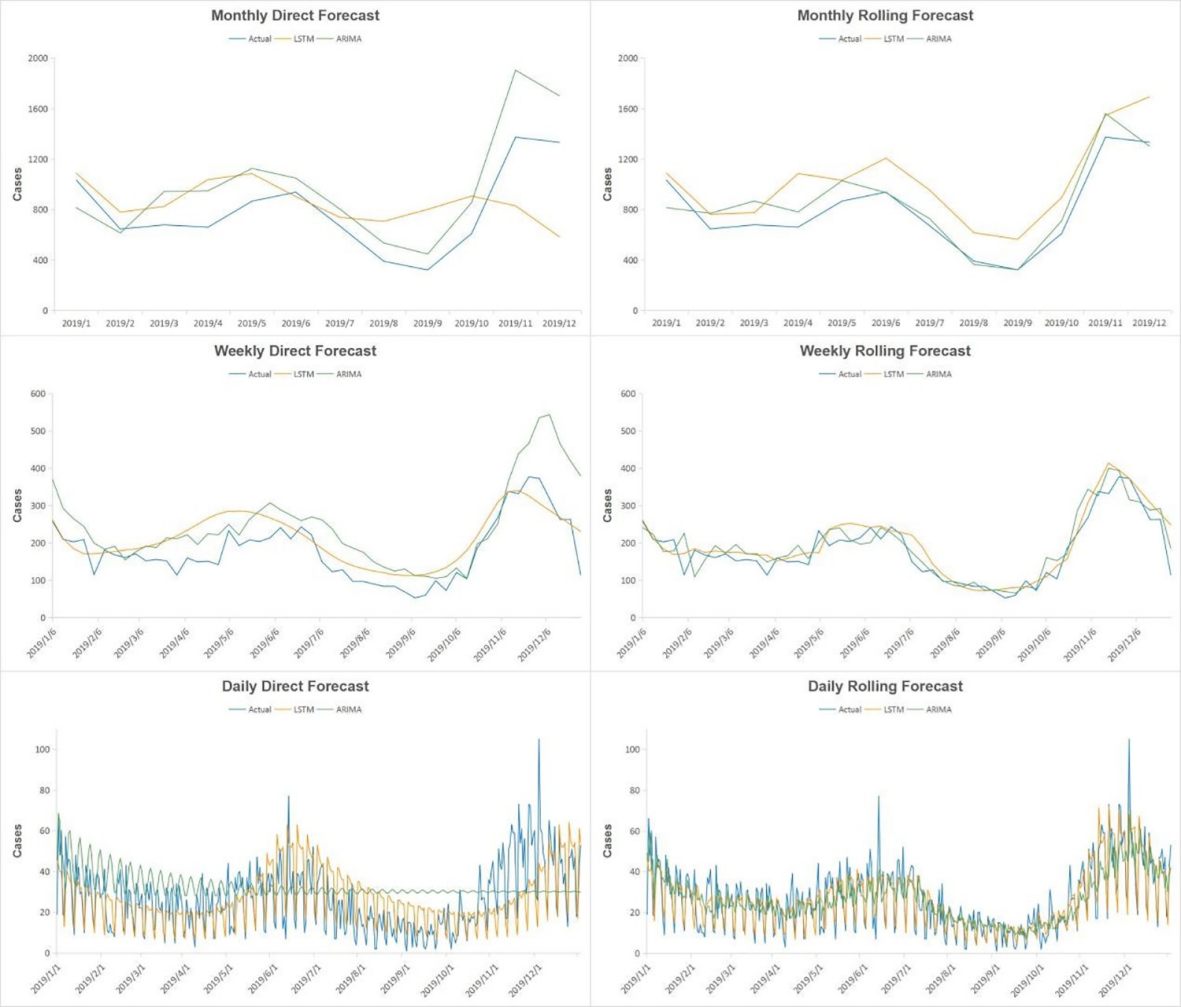
The observed hemorrhagic fever incidence and values predicted by ARIMA and LSTM models in 2019.

The best fitting model of ARIMA and LSTM were adopted to forecast the number of hemorrhagic fevers in 2019 in two different ways, direct forecasting and rolling forecasting. Predicted values were compared with the actual values to test the prediction effect of the models. RMSE, MAE and MAPE were applied to evaluate the prediction performance of the models and lower value means better performance. The results in Table 2 shows the values of RMSE, MAE and MAPE of the models. Those using rolling forecasting showed lower values than those of using direct forecasting for both ARIMA and LSTM, indicating the accuracy of the models using rolling forecasting was better than those using direct forecasting. ARIMA model produced lower error values than LSTM model in monthly and weekly series which indicated that ARIMA was more successful than LSTM for monthly and weekly forecasting. While the error values produced by LSTM were lower than those by ARIMA for daily forecasting in rolling forecasting model. Fig 4 shows the forecasting curves of ARIMA and LSTM models and the observed values in 2019. Both ARIMA and LSTM predicted the seasonal fluctuation well, particularly in rolling forecasting models.

**Table 2:** The forecasting performance of the two models.

Model		Direct forecasting			Rolling forecasting		
		RMSE	MAE	MAPE	RMSE	MAE	MAPE
**Monthly**	ARIMA (2, 1, 1) (0, 1, 1)_12_	205.55	152.77	13.13	108.38	72.67	8.51
	LSTM	354.95	284.92	43.17	247.53	224.42	34.2
**Weekly**	ARIMA (1, 1, 3) (1, 1, 1)_52_	60.07	44.44	17.59	31.09	20.56	11.85
	LSTM	54.18	44.10	33.21	35.98	26.21	17.81
**Daily**	ARIMA (5, 0, 1)	14.20	10.93	65.2	13.01	10.06	58.63
	LSTM	13.23	10.27	61.20	8.05	5.75	35.70

## Discussion

We adopted two of the most commonly applied models in infectious disease prediction to establish and compare forecasting models for monthly, weekly and daily incidence of hemorrhagic fever. This used the national communicable diseases monitoring data from 2013 to 2019 in China. Additionally, we optimized forecasting by using a rolling forecasting origin. To the best of our knowledge, this is the first study in China to build and compare different prediction models of hemorrhagic fever incidence using ARIMA and LSTM models with rolling forecasting origin and at different time scales. The results demonstrated that both ARIMA and LSTM could be used to build prediction models for the incidence of hemorrhagic fever, while different models might be suitable for incidence prediction at different time scales.

The incidence of hemorrhagic fever in China has had a slightly increasing trend in recent years. A large infected population and an increased social financial burden had been made due to large population bases in China, even with low incidence rates [31–33]. The results of our study showed that the incidence of hemorrhagic fever in China displayed a bimodal seasonal distribution during 2013 to 2019 as found in other studies [11, 34]. The highest peak was from autumn to winter and the second highest peak was from spring to summer. That may because rodents are more active and are more likely to have contact with people directly or indirectly during those two seasons. Incidence predictions may be of significance for prevention and control of hemorrhagic fever before an outbreak occurs. Our results provide great evidence for the prediction model building to establish an early warning system of hemorrhagic fever.

The patterns of incidence of hemorrhagic fever in China is suitable for the ARIMA model and LSTM model. However, the different principles of these two models resulted in different performances at different time scales. The principle of the ARIMA model is to filter out the high-frequency noise in the data, detect local trends based on linear dependence and predict the development trends [11]. Additionally, the ARIMA model transforms the influence factors of disease into some special time variables and then matches them. The limitation of this model is that ARIMA can only analyze the linear part of an infectious disease series [9]. However, the non-linear part of infectious disease data may not be white noise, meaning that some information may not be captured by the ARIMA model [11]. LSTM is an advanced kind of RNN and a deep learning application which is designed to learn temporal pattern, capture non-linear dependences and store useful memory for a longer time so produces better results in situations where the number of dataset is large [27]. In our study, according to Fig 1, the daily series had the largest number of data points and the most non-linear dependences while the monthly series had the smallest number of data points and the most linear dependences. The results show that ARIMA model tends to forecast more accurate results for which there is a clear trend in the series, whereas LSTM tends to do better on volatile time series with more instable components. In addition, ARIMA model and LSTM model have different requirements for sample size. ARIMA model needs statistical inference in the process of modeling, so it needs to meet the requirements of large samples. Studies have shown that ARIMA needed at least 50 historical statistics [35]. LSTM model is a complex neural network, and like any neural network requires a large amount of data to be trained on properly. Too few training samples will lead to over fitting. The larger the sample size, the higher the accuracy of the model [36]. If LSTM uses 64 or even 8 neurons, it can generate many parameters to be estimated. In this study, the monthly model and the weekly model had only 84 samples and 365 samples, respectively. It could be inferred that the LSTM model might be over fitted. Therefore, LSTM model is not recommended when the sample size is too small, such as monthly or weekly data. The above might be the reasons why ARIMA showed better performance in monthly and weekly predictions while LSTM displayed better performance in daily predictions.

One of the highlights of our study is that we forecasted the incidence of hemorrhagic fever in 2019 using the prediction models with “Rolling Forecasting Origin”, also called “walk-forward model validation”, which forecasts the incidence value by adding to the previously observed real incidence data solving the problem of connection actuality in the prediction phase of the method and increasing the accuracy of the prediction. In addition, we built models and compared the forecasting performances with three time scales including monthly, weekly and daily incidence. The results indicated that time scales should be taken into account when selecting prediction models of diseases because different models might be appropriate for disease forecasting at different time scales.

There are several limitations. Firstly, the data of this study was applied from the Public Health Science Data Center, which is based on the hospital reporting for the hemorrhagic fever monitoring cases. There may be selection or under-reporting bias. Secondly, only variation for hemorrhagic fever cases with time was considered due to data availability. The function of other possible impacting factors was ignored such as medical conditions and environment. Thus, the data should be continually updated to ensure high prediction accuracy and giving an accurate warning before hemorrhagic fever outbreaks. Thirdly, the hemorrhagic fever incidence data in this study was the total incidence in China, we cannot explore the province-specific performance of these models. The spatial factor is important for the development of hemorrhagic fever so the applicability of results in this study will need further exploration. Finally, there are many influencing factors of diseases, such as meteorological factors, individual differences, regional differences and so on [1, 2]. Therefore, in the future study, we plan to adopt ARIMA with exogenous input variables (ARIMAX) and multivariate-LSTM to build prediction models, which are based on the daily incidence of hemorrhagic fever and daily meteorological data.

## Conclusions

Both ARIMA and LSTM could be adapted to build prediction models for the incidence of hemorrhagic fever. While different models might be suitable for the incidence prediction at the different time scales. Rolling forecasting models performed better than direct forecasting models. Our results provide good references for future selection of prediction models and establishments of early warning systems for hemorrhagic fever.
